# Ourselves and Our Enterprise

**Published:** 1842-12

**Authors:** 


// THE WESTERN JOURNAL.

Vol. VI.—No. VI.

LOUISVILLE, DECEMBER 1, 1842.

### OURSELVES AND OUR ENTERPRISE.

This number completes the sixth volume of our Journal. It is with no ordinary feelings of regret, that, in looking back, we are compelled to admit that we have not made it equal, in variety and richness, to the expectations which its title, and the number of its editors, may have raised in the minds of its readers. But while making this confession, justice requires that we should remind them, and our brethren generally in the west, of their own delinquencies as writers; that in establishing a special department for their contributions, we had a right to expect their co-operation; and that one source of the barrenness to which we are confessing, is their own remissness. As we hope in future to do better ourselves, so we anticipate that they will display greater activity as contributors.

Our seventh volume will open with an elaborate experimental paper, by our indefatigable collaborator, Prof. Gross, on wounds of the intestines, illustrated with engravings. It will occupy a considerable portion of the original department of the first three numbers; and must, we think, be found acceptable to all who have a taste for patient and accurate inquiry.

A word to our correspondents. As two of us are often absent from Louisville during the vacations of our school, it is proper that communications for the Journal should be directed *To the Editors*, without naming any one, otherwise they may be sent away or remain unopened. In connexion with this suggestion, we wish to say that we have nothing to do with the business affairs of the Journal; and, therefore, that all letters relative to the loss of numbers in the mail, or to subscriptions, or enclosing money, should be directed to Messrs. *Prentice & Weissinger*, Publishers.

In conclusion, a hint to those who send us pamphlets. If any thing be written upon them, *the name, or respects, or any other salutations of the author or giver*, it subjects them to *letter* postage. Such is the present regulation of the Post Office Department; and, whether enforced generally or not, it is rigidly carried out in this city; whose Post Master, one of *our own profession*, has the integrity (of which we should feel proud) to execute, most faithfutly, the duties enjoined upon him by the government. EDS.

